# Supplementary figures and images for: Functional network modules overlap and are linked to interindividual connectome differences during human brain development
Source: PLoS Biol. 2024 Sep 18;22(9):e3002653. doi: 10.1371/journal.pbio.3002653 (PMC11441662; doi:10.1371/journal.pbio.3002653)

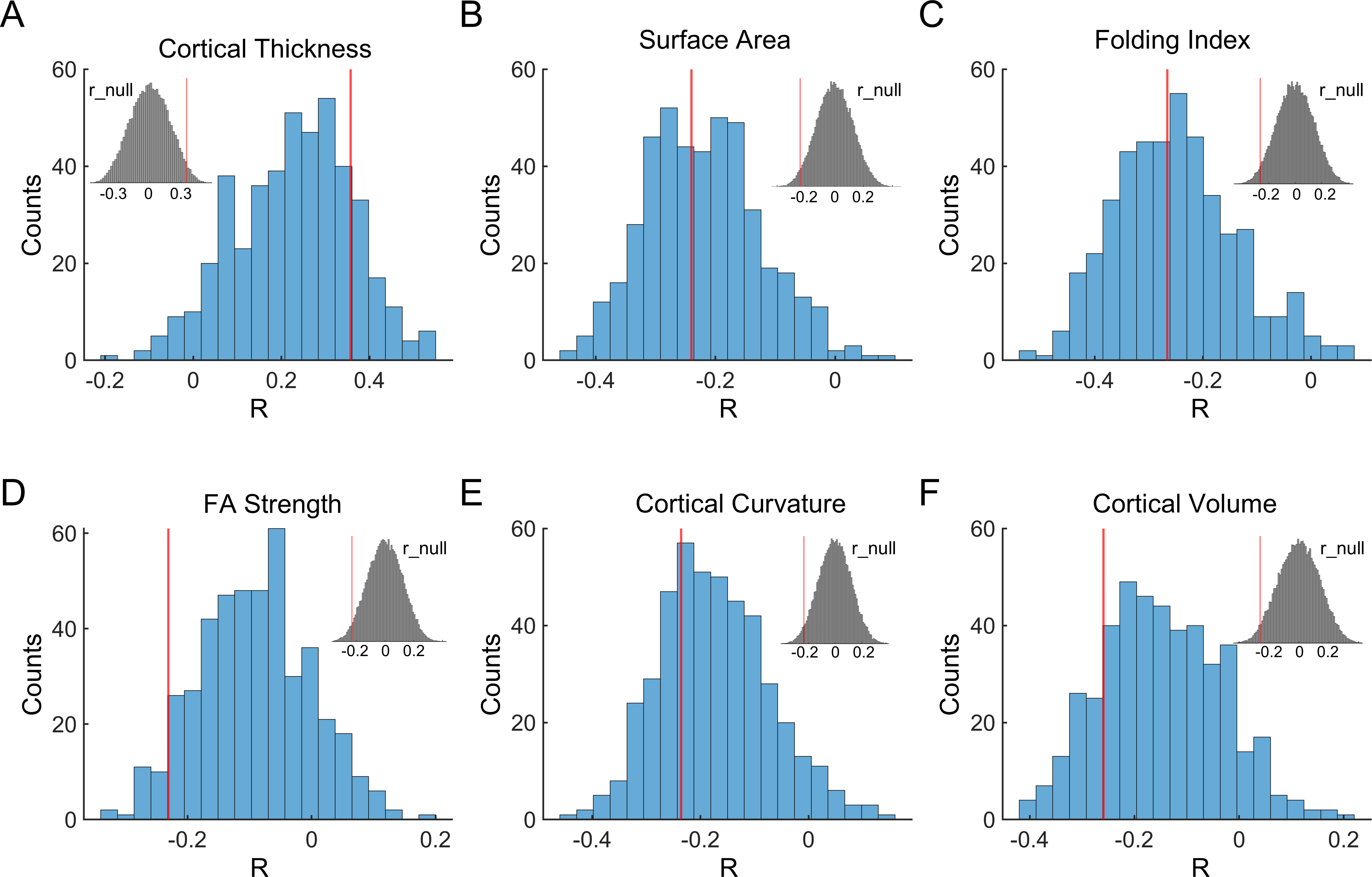

Supplement: S1 Fig — For each scan, we separately calculated Pearson’s correlation in spatial patterns between nodal entropy and each structural brain feature. (A) Frequency polygon of correlation coefficients for cortical thickness across rsfMRI scans. Compared to the null distribution, cortical thickness showed significant positive spatial correlations with nodal entropy in 17% of scans (74/446) (p < 0.05). (B) Frequency polygon of correlation coefficients for surface area in all rsfMRI scans. Compared to the null distribution, surface area showed significant negative correlations with nodal entropy in 44% of scans (196/446) (p < 0.05). (C) Frequency polygon of correlation coefficients for folding index in all rsfMRI scans. Compared to the null distribution, folding index showed significant negative correlations with nodal entropy in 46% of scans (207/446) (p < 0.05). (D) Frequency polygon of correlation coefficients for FA strength in all rsfMRI scans. Compared to the null distribution, FA strength showed significant negative correlations with nodal entropy in 5% of scans (24/446) (p < 0.05). (E) Frequency polygon of correlation coefficients for cortical curvature in all rsfMRI scans. Compared to the null distribution, cortical curvature showed significant negative correlations with nodal entropy in 30% of scans (136/446) (p < 0.05). (F) Frequency polygon of correlation coefficients for cortical volume in all rsfMRI scans. Compared to the null distribution, cortical volume showed significant negative correlations with nodal entropy in 17% of scans (76/446) (p < 0.05). In (A–F), the inset in the upper corner denotes the null distribution of the correlation coefficients. This null distribution was generated by aggregating the 100 permutation instances for each scan, resulting in a total of 44,600 permutation instances (446 scans × 100 times). For each permutation within each scan, a surrogate nodal entropy map was generated that preserved the spatial autocorrelation characteristics of [file pbio.3002653.s001.TIF]

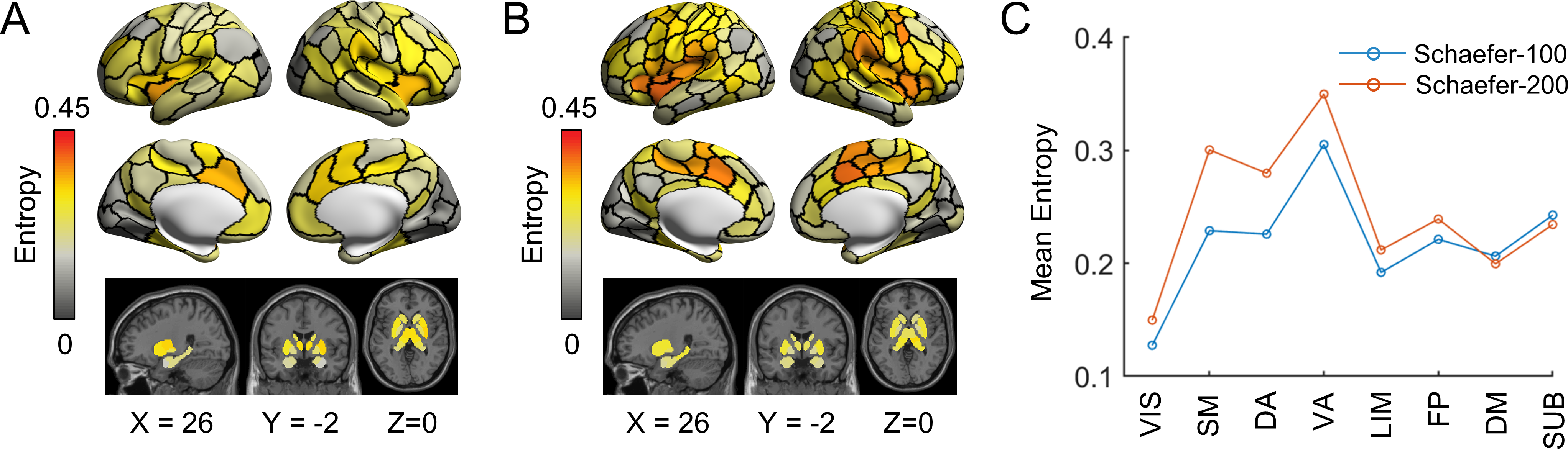

Supplement: S2 Fig — For the adult cohort, the overlapping modular architecture was separately detected in the group-level functional networks obtained from different functional parcellations. (A) Nodal overlap in the functional networks with a coarse parcellation. This network comprised 100 cortical nodes obtained from the Schaefer-100 atlas [97] and 32 subcortical regions [49]. (B) Nodal overlap in the functional networks with a fine parcellation (i.e., main results). This network comprised 200 cortical nodes obtained from the Schaefer-200 atlas [97] and 32 subcortical regions [49]. (C) Extent of nodal overlap for 8 systems at different spatial resolutions. Similar distributions of nodal overlap were observed between the 2 parcellations. VIS, visual; SM, somatomotor; DA, dorsal attention; VA, ventral attention; LIM, limbic; FP, frontoparietal; DM, default-mode; SUB, subcortical. (TIF) [file pbio.3002653.s002.TIF]

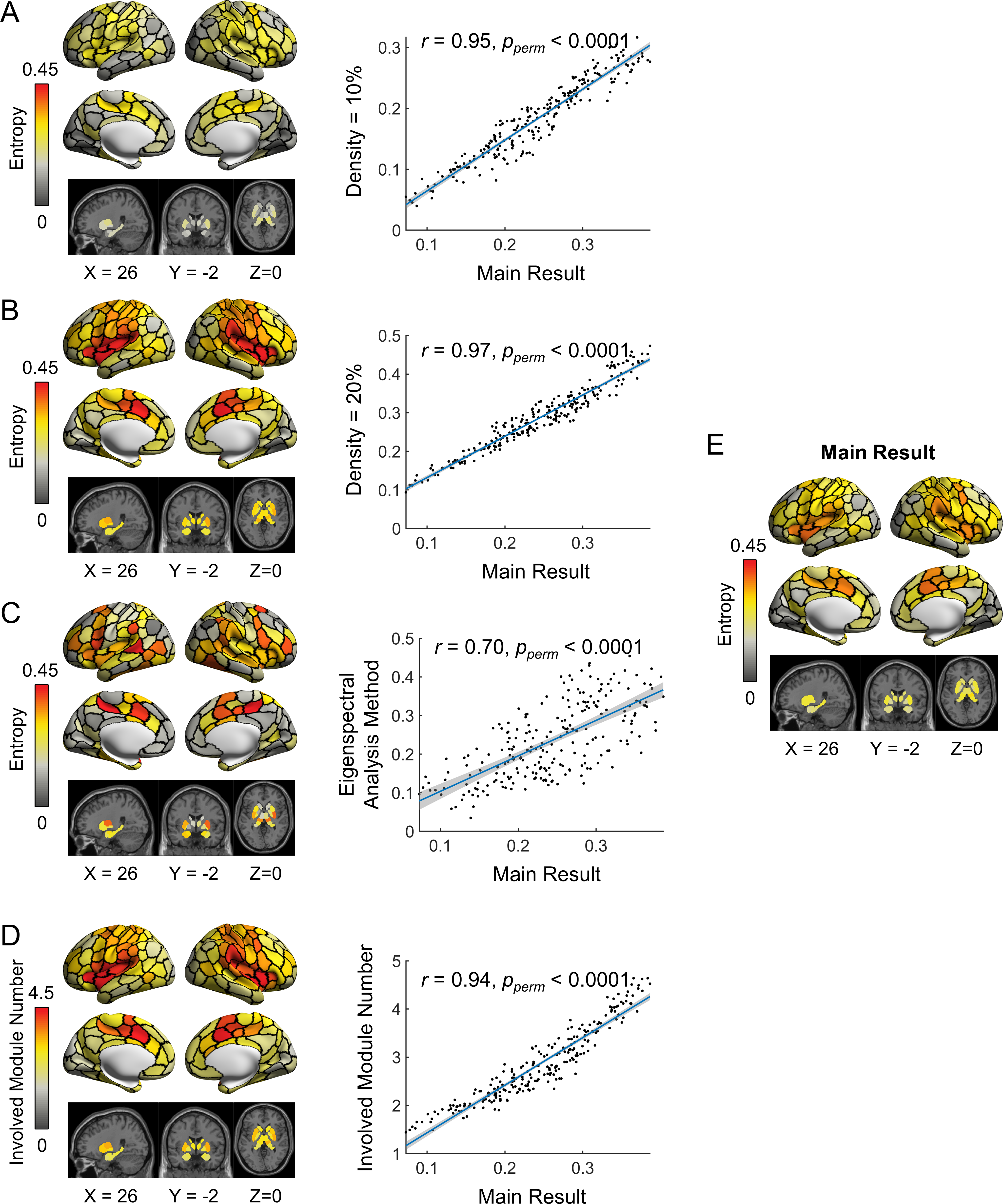

Supplement: S3 Fig — All of these analyses were specified to the group-level functional network for the adult cohort. (A) Network density of 10% for functional network construction. (B) Network density of 20% for functional network construction. (C) Eigenspectral analysis for module detection in the edge graph. (D) Number of involved modules was used to quantify the extent of node module overlap. (E) Main result as a reference. In each case, all the network construction and analysis strategies were set to be the same as those in the main analysis, except for the strategy of interest. All correlations were assessed with Pearson’s correlation across nodal regions. The significance of spatial similarity was assessed by comparing the observed value to a null distribution generated through 10,000 permutations that retained the spatial autocorrelation characteristics of the nodal entropy map of the original adult cohort in the main results. (TIF) [file pbio.3002653.s003.TIF]

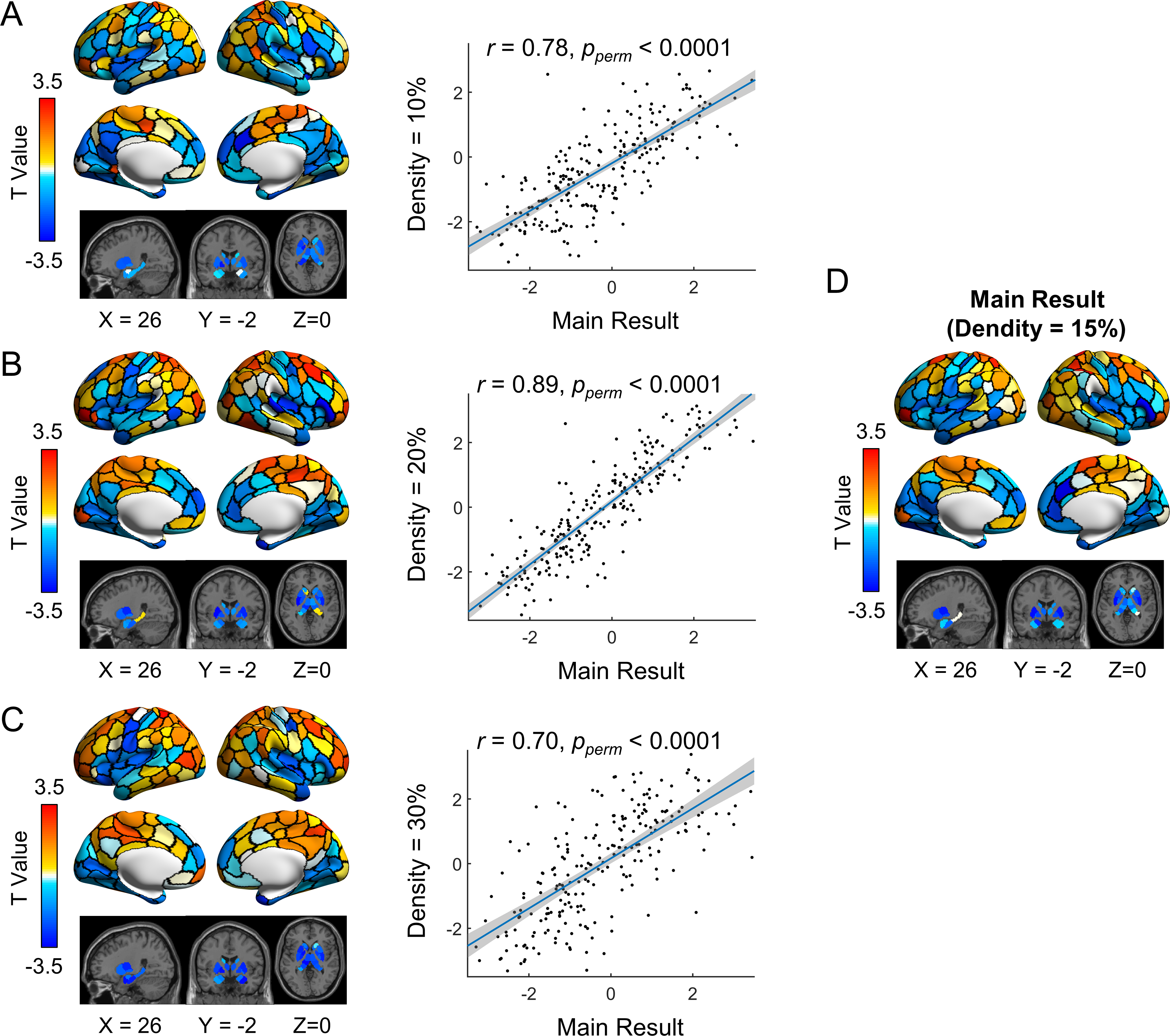

Supplement: S4 Fig — For each rsfMRI scan of children, the brain functional network was generated with different network thresholding strategies. (A) Network density of 10% for functional network construction. (B) Network density of 20% for functional network construction. (C) Network density of 30% for functional network construction. (D) Network density of 15% (i.e., main result as a reference). In each case, all the network construction and analysis strategies were set to be the same as those in the main analysis, except for the strategy of interest. All correlations were assessed using Pearson’s correlation across nodal regions. The significance of spatial similarity was assessed by comparing the observed value to a null distribution generated through 10,000 permutations that retained the spatial autocorrelation characteristics of the original age effect map (t-value map) in the main results. (TIF) [file pbio.3002653.s004.TIF]

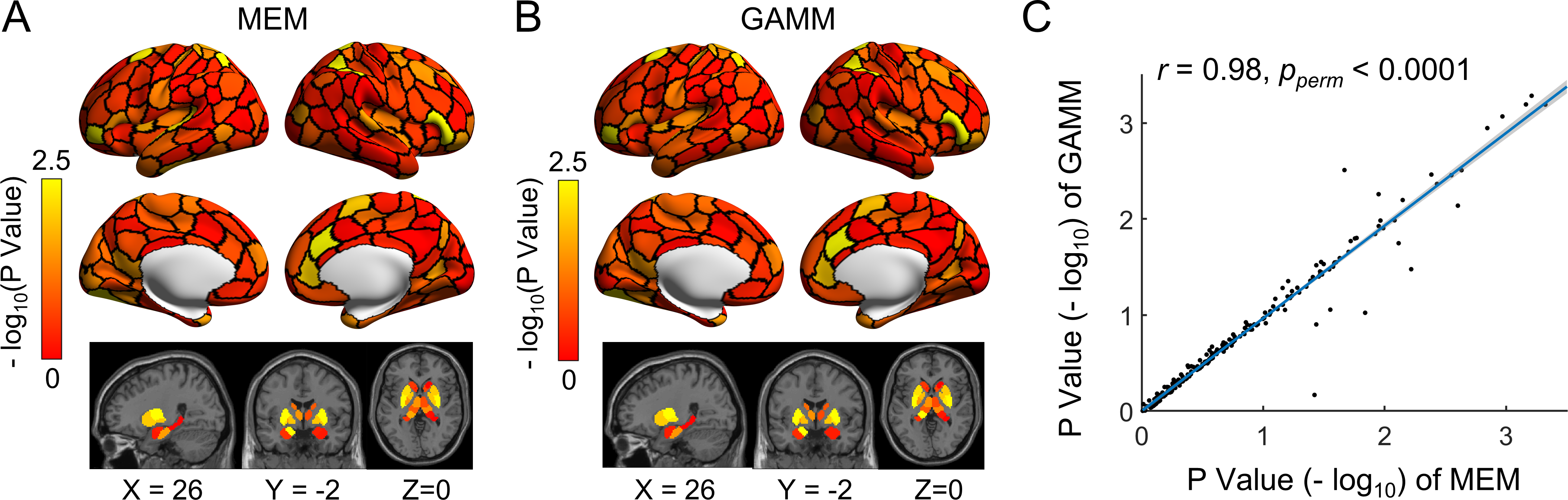

Supplement: S5 Fig — (A) Age effects on nodal entropy using a mixed effects model. (B) Age effects on nodal entropy using a generalized additive mixed effects model. (C) Spatial correlation of age effects on nodal entropy between 2 statistical methods. The significance of spatial similarity was assessed by comparing the observed value to a null distribution generated through 10,000 permutations that maintained the spatial autocorrelation characteristics of the original age effect map (i.e., p-value map) obtained from the MEM in the main results. MEM, mixed effects model; GAMM, generalized additive mixed model. (TIF) [file pbio.3002653.s005.TIF]
