# Supplementary material for: Functional network modules overlap and are linked to interindividual connectome differences during human brain development
Source: PLoS Biol. 2024 Sep 18;22(9):e3002653. doi: 10.1371/journal.pbio.3002653 (PMC11441662; doi:10.1371/journal.pbio.3002653)
Supplement: Attachment — Submitted filename: ResponsesToReviewers.docx [file pbio.3002653.s007.docx]

**Reviewer #1 (Adam Pines):**

This is a nice paper with several beneficial contributions. The description of the need for studying overlap in networks is very well-articulated, as are the methods. The longitudinal design of the study is another advantage, and the publicly available code is a clear strength. I do have several comments, which are listed below. I'm suggesting major revisions, but I think they are minor as far as major revisions go.

**R:** We thank you for the positive comments and constructive suggestions.

**Major Comments:**

1.1 Figure 5 is interesting, but these correlations are quite low, particularly for brain maps. Please use something like you did for figure 6 (i.e., Burt et al 2020 or the spin test for parcels) to correct brain map associations by accounting for spatial autocorrelation. It's not clear that any of these associations are above chance, after accounting for spatial autocorrelation.

**R:** According to your suggestion, we assessed the significance level of spatial similarity using permutation tests that corrected for spatial auto-correlation (Burt et al., NeuroImage 2020). In the revised manuscript, we have updated the relevant Methods and Results section.

The relevant Methods section (Page 22):

“The significance level of each spatial similarity was assessed using permutation tests that corrected for spatial autocorrelation [56]. Specifically, given a cognitive term of interest, we constructed a null distribution of correlation coefficients for each percentile bin separately based on 10,000 permutations. For each permutation, we generated a surrogate map preserving the spatial autocorrelation of the original age effect map, and then calculated the association with that specific cognitive term for each percentile bin. We further compared each empirically observed value to the corresponding null distribution to derive the significance level.”

The relevant Results section (Page 10):

**Figure 5. Cognitive function decoding of brain regions.** Nodal regions were sorted in descending order according to developmental changes in the functional module overlap. The results were obtained based on the information in the NeuroSynth meta-analytic database [55]. The significance level of the spatial similarity was assessed using permutation tests (*n* = 10,000) that corrected for spatial autocorrelation [56]. *, *p_perm_* < 0.05; **, *p_perm_* < 0.01; ***, *p_perm_* < 0.001.

1.2 Please clarify if entire subjects are held out of train-test in figure 6. It is essential to not include the same subjects (across different observations) across train-test splits to avoid leakage.

**R:** Thank you for this valuable suggestion. To avoid the possibility of data leakage that could occur by including longitudinal scans from the same subjects between the training and test sets, we reperformed the age prediction analysis based on 305 rsfMRI scans that were randomly selected from independent subjects. Specifically, for children with longitudinal data, only one rsfMRI scan was randomly selected from each individual and the other scans were discarded. We then performed the ten-fold cross-validation based on these 305 independent samples. To reduce potential bias, we repeated the prediction analysis 1,000 times with random scan selection, resulting in a distribution of prediction accuracy. Accordingly, to assess the contribution of nodal features to the prediction, the prediction weights of nodal regions were obtained by averaging the nodal weights over the 1,000 times of random sampling.

The relevant Methods section (Page 22):

“To avoid the possibility of data leakage that could occur by including longitudinal scans from the same children in both the training and test sets, we performed the ten-fold cross-validation using 305 rsfMRI scans that were selected from independent subjects. For children with longitudinal scans, only one scan was selected from each individual and the other scans were discarded. To reduce the potential bias, the ten-fold cross-validation analysis was repeated 1,000 times with random scan selection.”

“To determine the contribution of nodal features to the prediction model, we trained another SVR model using all the 305 scans to improve the estimation accuracy [104, 105]. The resulting regression coefficients were regarded as the weights denoting the importance of all features. The final contribution weights of nodal regions were obtained by averaging the estimated weights over the 1,000 times of random sampling.”

The relevant Results section (Page 10):

“To avoid the possibility of data leakage that could occur by including scans from the same children in both the training and test sets, a total of 305 rsfMRI scans from independent subjects were selected for use in the prediction analysis. For a given individual with longitudinal scans, only one scan was randomly selected from the individual. The age prediction analysis was repeated 1,000 times with random scan selection. We found that the spatial patterns of nodal entropy significantly predicted individual chronological age (*r* = 0.14 ~ 0.52, all *p_perm_*s < 0.05; Figure 6B). The significance level of the prediction accuracy was assessed with permutation tests (*n* = 10,000) in which the original age was shuffled across children. Regions with high contributions were primarily located in the dorsal attention, ventral attention, and default-mode systems (Figure 6C). Furthermore, we found that the nodal contribution weights showed a significant positive correlation with the developmental changes in nodal entropy in terms of age-related *t* values (Pearson’s correlation *r* = 0.48, *p_perm_* < 0.0001; Figure 6D).”

**Figure 6. Age prediction based on spatial patterns of nodal overlap. (A)** Schematic representation of the SVR prediction model based on ten-fold cross-validation. **(B)** Frequency polygon for age prediction accuracies using the ten-fold SVR model for 1,000 times of randomly selected samples. For each time, rsfMRI scans were randomly selected from 305 independent subjects. The gray frequency polygon in the inset displays the null distribution of prediction accuracies based on the permutation tests (*n* = 10,000) by randomly sampling the scans and shuffling the original ages across the scans. The red line indicates the significant level (*p* < 0.05). **(C)** Spatial distributions of the nodal contribution in the prediction model. Left: Contribution weight at the regional level. Right: Contribution weight at the system level. The nodal contribution weights were obtained by averaging the contribution weights across 1,000 times of randomly selected samples. Positive and negative weights were separately averaged within each system. **(D)** Left: Spatial pattern of age effect of on nodal overlap in terms of *t* values. Right: Nodal contribution weight shows a significant positive correlation with the development of nodal overlap based on Pearson’s correlation analysis. The significance level of the similarity was assessed using the permutation tests (*n* = 10,000) to correct for spatial autocorrelation [56]. Pos, positive; Neg, negative; VIS, visual; SM, somatomotor; DA, dorsal attention; VA, ventral attention; LIM, limbic; FP, frontoparietal; DM, default-mode; SUB, subcortical; SVR, support vector regression.

**1.3** The multimodal and cognitive aspects are good efforts but are a bit oversold. They might be interesting enough to warrant inclusion as either a main text or supplemental figure, but I don't think its fair to say the authors have "provided novel insights into the neural mechanism underlying individual cognitive development.", especially because the structure-function association is multivariate rather than tractable to a more straightforward univariate correspondence. What are the mechanisms, and furthermore, where are the individual-level measures of cognition? Please clarify or temper these claims.

**R:** Thank you for pointing out these issues. In the revised manuscript, we have rephrased relevant descriptions throughout the manuscript, including removing the description of “neural mechanism underlying individual cognitive development”.

The relevant Discussion section (Page 13 and 15):

“These findings highlight the gradual maturation of the overlapping modular architecture and its potential structural substrates during childhood and adolescence, providing novel insights into the developmental rules of the human brain.”

“Of note, during the adult lifespan, the age-dependent overlapping functional modules are associated with fluid cognition [40]. Further exploration is warranted regarding the potential relationship between the maturation of the overlapping functional modules and individual cognitive development.”

**1.4** Maximum head motion > 3 mm or 3° or mean framewise displacement (mFD) > 0.5 mm: seems like an incredibly liberal threshold. Could the authors provide a surface/parcel plot of entropy associations with FD parallel to their surface/parcel plot of entropy associations with age? I would imagine there are a ton of residual motion effects, and it would be useful for the reader to know whether or not this is the case, particularly if they can compare effect sizes/prominence relative to those reported for age.

**R:** Thank you for raising this important concern. In this study, we used these criteria to exclude rsfMRI scans with extensive absolute or relative head motion. For 491 rsfMRI scans used in the main analysis, the mean FD (mFD) for different scans ranged from 0.05 mm to 0.40 mm (mean ± SD = 0.17 ± 0.07). To mitigate the influence of head motion on our analysis, we have applied the following strategies: (i) During data preprocessing, we included 24 head motion parameters, the global brain signal, and “bad” time points (FD > 0.5 mm) as covariates in the nuisance regression, which is considered an effective head motion correction strategy (Ciric et al., NeuroImage 2017; Parkes et al., NeuroImage 2018). (ii) The head motion parameter (i.e. mFD) was included as a covariate in the mixed effects model for age effects estimation (Ciric et al., NeuroImage 2017; Cui et al., Neuron 2020).

According to your suggestion, we conduced additional validation analyses to further explore the potential influence of head motion on developmental effects. First, we calculated the correlation between mFD and age across subjects, and observed a significant but weak correlation (*r* = -0.09, *p* = 0.05) (Figure S6A). Second, the influence of age and head motion on nodal entropy was assessed using the mixed effects model employed in the main analysis. In this model, age was designated as the independent variable, while the head motion parameter (i.e. mFD) was included as a covariate. The effects of age and head motion on nodal entropy were displayed as different *t*-value maps (Figure S6B). Finally, for a more stringent control of head motion, we further excluded subjects with mFD > 0.2 mm and re-estimated the age effects using the mixed effects model. The age-related *t*-map showed high spatial correlation with the main results (*r* = 0.89, *p_perm_* < 0.0001) (Figure S6C). These results suggest that head motion has a limited effect on our main findings of developmental changes in nodal entropy.

In the revised manuscript, we have provided additional analyses and revised the discussion of head motion to better illustrate the potential effects of head motion.

The relevant Methods section (Page 24):

“To assess the robustness of the developmental effects of node entropy, we examined the potential impact of the network thresholding strategy, the statistical model for estimating age effects, and the head motion control strategy. …… Third, to assess the potential effect of head motion, we performed a more stringent head motion control by excluding rsfMRI scans with mFD > 0.2 mm. Age effects were assessed using the mixed effects model based on the remaining scans.”

The relevant Results section (Page 13):

“Furthermore, a more stringent head motion control strategy was implemented by excluding rsfMRI scans with mFD > 0.2 mm (retaining 355 scans from 233 children). The age effect map exhibited a high degree of spatial correlation (*r* = 0.89, *p_perm_* < 0.0001) with the main results (Figure S6). These sensitive analyses indicate that the age effects on nodal entropy were robust across methodological variations.”

The relevant Discussion section (Page 15 and 16):

“First, inner-scan head motion introduces a spatially inhomogeneous bias in functional connectivity estimation, which is age dependent [76]. To reduce the potential influence of head motion, we excluded rsfMRI scans with extensive head motion and further included 24 head parameters, global brain signals, and “bad” time points during nuisance regression [77, 78]. The individual head motion parameters (i.e. mFD) were also included in the mixed effects model when assessing the age effects [77, 79]. When performing a more stringent head motion control, the developmental effects of nodal entropy remained almost unchanged. These results suggest that the head motion has a limited effect on our findings. Nevertheless, it would be valuable to further refine head motion correction methods to mitigate the residual influence of head motion on functional network development.”

The relevant Supplementary materials section (Page 38):

**Figure S6. Effects of head motion parameters on developmental changes of nodal entropy. (A)** Age effects on head motion parameters. Pearson’s correlation analysis revealed that individual age and mFD values showed a significant but weak correlation. **(B)** Left: Age effects on nodal entropy. Right: Head motion effects on nodal entropy. The influence of age and head motion on nodal entropy was evaluated using a mixed effects model. In this model, age was designated as the independent variable, while the head motion parameter (i.e. mFD) was included as a covariate. The variable of sex was also included in the model as a covariate. **(C)** Left: Spatial pattern of age effects on nodal module overlap with a head motion control strategy of mFD < 0.5 mm (main results). Middle: Spatial pattern of age effects on nodal module overlap with a more strict head motion control strategy with mFD < 0.2 mm. Right: Spatial correlation of age effects on nodal entropy between two head motion control strategies. The significance of the spatial similarity was assessed by comparing the observed value to a null distribution generated through 10,000 permutations that retained the spatial autocorrelation characteristics of the original age effect map (*t*-value map) in the main results. mFD, mean framewise displacement.

**Minor Comments:**

**1.5** The authors appear to make two claims of primacy that are unsubstantiated. Despite citing work from the same lab, I assume the authors were unaware of our study on overlapping functional modular organization in children and adolescents when they claimed "our study is the first to uncover overlapping functional modular organization in children and adolescents" (See Pines et al., Nature comms, 2022). We also take a personalized approach, directly tie individual level cognitive effects in, developed a generalized estimating equations approach to run stats on individual locations belonging to multiple networks, and explicitly quantify network edge development as a function of the hierarchical similarity (hierarchical distance) as alluded to by the current authors in this manuscript's discussion. You are not obligated to cite us, but the claim of primacy is false and should be removed. Further, although not in developing individuals, this paper is similar enough to "Edge-Community Entropy is a Novel Neural Correlate of Aging and Moderator of Fluid Cognition", now out in J Neurosci, where that work probably should be cited.

The second unsubstantiated claim of primacy, although indirect, is "However, until now, there has been no gold standard for evaluating the quality of the detected overlapping functional modular structure in the human brain." I'm not sure the authors meant it this way, but it sounds like they are implying that they pioneered an approach to evaluate the quality of overlapping functional modular structure in the human brain. Unless I missed something substantial in the paper, this appears to be taking credit for the development of the approach, which by my understanding was done by the Betzel lab (Faskowitz et al), not the current authors. Apologies if I'm misunderstanding this claim, but I would at least clarify it.

**R:** We thank you for pointing out these issues and providing the references. In the revised manuscript we have cited the suggested references in the revised manuscript and further clarified the differences between our work and several related studies (including Pines et al., Nat Commun 2022 and Shankar et al., J Neurosci 2024). Specifically, our work has primarily focused on quantifying multiple modular affiliations of brain regions in children and adolescents with the approach of overlapping functional modules, and investigating the developmental rules of the overlapping functional modules during childhood and adolescence. Regarding the second issue, we have rephrased the relevant descriptions in the revised manuscript to make this point clear.

Regarding the first issue, the relevant Discussion section (Page 13 and 14):

“The functional network exhibits an intricate community structure with multi-scale organization in both adults [57] and youths [58], and its spatial topography varies across individuals [21, 36, 37]. In this study, we used the novel framework of overlapping modular architectures, which provides an intuitive representation of the functional interactions between modules and the multiple roles of brain regions [59, 60] and deepens the understanding of the organizational principles of the human brain [31-35]. Compared to previous studies on overlapping modules in adults [31-35] and the elderly [40], our research focuses on the development of overlapping functional modular organization during childhood and adolescence.”

“We found that each functional module comprised nodal regions involved in at least two prior functional systems, regardless of the age subgroup. These systems contained in the same functional module have usually been found to be located in adjacent functional gradients [50]. This may be partially attributable to the fact that regions locating in proximate functional hierarchy tend to show strong functional interactions [58].”

Regarding the second issue, the relevant Discussion section (Page 15):

“Previous studies on overlapping modules in the human brain have primarily used node-centric methods [32, 33, 35, 39], usually assuming probabilistic membership or maximal clique for nodes, which struggle to capture intricate overlapping configuration [72]. In this study, we employed an edge-centric algorithm [30, 44] to identify the overlapping modular architecture for brain nodes, offering an intuitive and detailed representation of multiple module affiliations of nodes [30, 34]. A recent study suggests that the overlapping modular architecture revealed by the edge-centric approach can be explained by surrogate interedge similarity derived from the node-level functional connectivity [73]. Further research is needed to explore the differences between these two approaches.”

**1.6** Reliance on linear models might be one reason the authors are not returning a ton of significant results in figure 3. There's every reason to believe development is non-linear in this age range, as many of the authors' citations point to (see citation 9 for a particularly compelling example). I think the results they returned are real, but choice of linear mixed effect models seems like a suboptimal fit to the data at hand compared to something like generalized additive mixed effect models. It's not 100% necessary for this paper to make a contribution to the literature, but seems like a clear step forward.

**R:** In this study, we have employed both linear and quadratic models to assess age effects on each brain measure of interest. The optimal model was selected for each brain measure based on the Akaike Information Criterion (AIC). We found that all significant results identified were selected from the linear model. Following your suggestion, we also used the generalized additive mixed model to explore potentially complex, nonlinear effects. This model was implemented in R using the package mgcv. We found that the spatial patterns of age effects were highly similar to the main results in terms of significance levels (Figure S5). The relevant methods and results have been provided in the revised manuscript.

The relevant Methods section (Page 24):

“To assess the robustness of the developmental effects of node entropy, we examined the potential impact of the network thresholding strategy, the statistical model for estimating age effects, and the head motion control strategy. …… Second, we used the generalized additive mixed model (GAMM) [111] to assess the potentially complex nonlinear age effects. This model was implemented in R using the package mgcv.”

The relevant Results section (Page 13):

“Subsequently, the development of nodal entropy was validated by employing different network thresholding strategies (i.e. 10%, 20%, and 30%) (Figure S4) and the generalized additive mixed model for estimating age effects (Figure S5). The spatial patterns of age effects on nodal entropy remained highly similar to the main results, with spatial correlations ranging from 0.70 to 0.98.”

The relevant Supplementary materials section (Page 37):

**Figure S5. Spatial patterns of developmental changes in nodal entropy using different statistical models. (A)** Age effects on nodal entropy using a mixed effects model. **(B)** Age effects on nodal entropy using a generalized additive mixed effects model. **(C)** Spatial correlation of age effects on nodal entropy between two statistical methods. The significance of spatial similarity was assessed by comparing the observed value to a null distribution generated through 10,000 permutations that maintained the spatial autocorrelation characteristics of the original age effect map (i.e. *p*-value map) obtained from the MEM in the main results. MEM, mixed effects model; GAMM, generalized additive mixed model.

**1.7** I would recommend describing what went up and down in the sentences: "Statistical analysis revealed that seven nodal regions showed significant linear changes with age (pFDR corrected < 0.0014, Figure 3C). These regions showed dissociable age-related changes, with significant increases mainly located in the superior and inferior parietal lobules and the lateral prefrontal cortex and significant decreases mainly located in the ventral and medial prefrontal cortex and the putamen." I assume it's entropy but that's never actually stated in results or the figure caption.

**R:** You are correct. In the revised manuscript, we have clarified this issue in the Results section.

The relevant Results section (Page 7):

“Statistical analysis revealed that nodal entropy in seven nodal regions showed significant linear changes with age (FDR-corrected *p* < 0.05, Figure 3C).”

**1.8** Could the authors allocate a sentence or two to describing why they are fitting age as a random effect rather than purely as a fixed effect with random effects for "subname"?

**R:** In the mixed effects model used here, the age effects were split into two parts, including a fixed effect and a random effect. The fixed effect accounts for population-level age effects, while the random effect accounts for individual variability in the age-related slope. The addition of the random age effect allows the model to capture individual differences in developmental trajectories, thus providing a more accurate estimate of developmental changes. The fixed age effect was used to report developmental changes in the brain measure. We have added some explanations in the revised manuscript to clarify this issue.

The relevant Method sections (Page 21)：

“In these equations, *y_ij_* represents the brain measure of subject *i* at the *j*th scan, *b_i_* represents individual-specific intercept, $\beta_{age}$ represents the fixed effect, *b_age,i_* represents the random effect, and $\varepsilon_{ij}$ represents the residual. The fixed effect $\beta_{age}$ accounts for the population-level age effects, while the random effect *b_age,i_* accounts for individual variability in the age-related slope. The addition of the random effect allows the model to capture individual differences in developmental trajectories, thus providing a more accurate estimation of developmental changes. The fixed age effect was used to capture the developmental changes in the brain measure.”

**1.9** I might avoid using the phrase "brain age", as this paper does not seem to be about discrepancies between predicted and actual age (which is a good thing!). Brain age literature is rife with problems (see Pitfalls in brain age analyses. Human brain mapping) that this current paper is not subject to.

**R:** In the revised manuscript, we have rephrased all the terms of "brain age" as "age" or "chronological age".

**1.10** It's not clear that you aren't using topological and topographical interchangeably. It's largely a stylistic choice to be consistent with that or not, and clarifying or standardizing your usage of these words is not something that will change my accept/non-accept opinion. However, some believe these refer to entirely different constructs (topology being purely in graph-space, topography representing a spatial distribution).

**R:** We fully agree that these two terms refer to different concepts. In our manuscript, we have used the topography to refer to the spatial distribution of brain measures, and we have updated the terminology throughout the text to ensure consistency.

**1.11** This is also just my opinion and not something. I would suggest rejecting this paper for, but I don't think this reasoning is entirely coherent: "The high nodal overlap in the sensorimotor regions indicates the functional diversity of this system, which is consistent with its involvement in multiple modules (Figure 2E). In addition to the widely known sensorimotor functions [54], these regions may be involved in other cognitive functions or behaviors, such as attention allocation [55], speech perception [56], and emotional regulation". In many papers, including the Margulies PNAS paper cited several times, there is a clear case for sensorimotor regions being the most specialized for one specific modality out of all cortical regions (i.e., unimodal). The authors are by no means obligated to take the same explanation we have in similar papers (Pines et al., Nature comms, 2022, Luo et al., Nature comms, 2024), but as is I find their logic inconsistent with the citations they invoke.

**R:** Thank you for providing these constructive suggestions and the relevant references. In the revised manuscript, we have rephrased the relevant discussion and interpreted the high nodal entropy in the sensorimotor regions in terms of between-network coupling (Pines et al., Nat Commun 2022; Luo et al., Nat Commun 2024) and nested multi-scale organization (Betzel and Bassett, NeuroImage 2017; Power et al., Neuron 2011). The high nodal entropy in these regions indicate that the sensorimotor regions may be involved in different cognitive processes, which warrants further investigation.

The relevant Discussion section (Page 14):

“The high nodal overlap in the sensorimotor regions may indicate a high level of between-module integration of this system, which aligns with previous findings that sensorimotor networks exhibit greater between-network coupling in comparison to association cortices [58, 62]. The high nodal entropy in these regions may be attributed, in part, to their tendency to exhibit nested multi-scale organization, as demonstrated in previous studies [63]. Despite the highly specialized functions of sensorimotor regions [50, 60], the potential functional significance of the high overlap in these regions warrants further investigation.”

**1.12** Edge-centric analyses are an important attempt to get at different aspects of network organization, but are subject to important limitations. Please consider the work "A mathematical perspective on edge-centric brain functional connectivity" in nature comms in either your introduction or discussion.

**R:** In the revised manuscript, we have added some discussion of the limitations of edge-centric analyses.

The relevant Discussion section (Page 15):

“Previous studies on overlapping modules in the human brain have primarily used node-centric methods [32, 33, 35, 39], usually assuming probabilistic membership or maximal clique for nodes, which struggle to capture intricate overlapping configuration [72]. In this study, we employed an edge-centric algorithm [30, 44] to identify the overlapping modular architecture for brain nodes, offering an intuitive and detailed representation of multiple module affiliations of nodes [30, 34]. A recent study suggests that the overlapping modular architecture revealed by the edge-centric approach can be explained by surrogate interedge similarity derived from the node-level functional connectivity [73]. Further research is needed to explore the differences between these two approaches. Of note, identifying the overlapping modular architecture using the current edge-centric method faces several challenges. First, uncertainties remain regarding whether and how the functional correlation matrix is thresholded to construct a stable node-level functional network [74, 75]. Second, this study only considers the relationships between edge pairs that share common nodes, neglecting potential indirect relationships between edges. Higher-order relationships between edges could be considered in future studies.”

**1.13** I can't find a protocol paper or more detail on it, but it's odd that a HARDI scan would have ordinary diffusion weightings only. Would the authors mind confirming that only b=0 and b=1,000 were used? Or citing a protocol paper if one exists?

**R:** Thank you for raising this concern. In this study, we used a 64-direction single-shell high angular resolution diffusion imaging (HARDI), which included two b values (i.e. b=0 and b=1,000), rather than the more complex multi-shell scheme. There are two main reasons for this design. First, the 64 directions go beyond traditional diffusion tensor imaging (DTI) and can be applied to higher order diffusion models. The high angular resolution allows more accurate detection of diffusion variations along different directions, thus resolving multiple fiber orientations within a single voxel and improving the accuracy of tractography. Secondly, this protocol takes into account acceptable scanning time for children and adolescents. In the revised manuscript, we have clarified this issue and provided the relevance references.

The relevant Method section (Page 17):

“*(iv) Diffusion MRI.* The scans were acquired using a single-shell high angular resolution diffusion imaging (HARDI) sequence [83, 84]: TR = 7500 ms, TE = 64 ms, flip angle = 90°, FOV = 224 × 224 mm^2^, acquisition matrix = 112 × 112, slice number = 70, slice thickness = 2 mm, and bandwidth = 2030 Hz/Px. The complete sequence consisted of 64 diffusion-weighted directions (b-value = 1000 s/mm^2^) and 10 non-diffusion-weighted directions (b-value = 0 s/mm^2^). The use of multiple gradient directions allows more accurate detection of diffusion variations along different directions [83, 84], thus resolving multiple fiber orientations within a single voxel and improving the accuracy of tractography.”

**1.14** "Then, a weighted functional network was obtained by thresholding the correlation matrix with a density of 15% (i.e., 4020 edges) to exclude potential spurious or weak correlations." This seems antagonistic to the idea of analyzing overlapping associations, which as the authors show, often fall into secondary or tertiary "network memberships" or more broadly FC strengths. Given that the data is already heavily reduced to 232 parcels, there doesn't seem to be any computational need for this step. Can the authors justify this choice? I do appreciate the inclusion of S2A and S2B to show similarity in the relative (although not absolute) distribution of entropy across regions across thresholds.

**R:** As the reviewer noticed, the estimation of module affiliations of a brain node depends on the module assignment of all the edges of that node. Thus, the accurate and stable estimation of the edges of interest and the inter-edge association is crucial for the subsequent overlapping modules analysis. Previous studies suggest that weak and non-significant functional correlations may represent spurious connections and tend to obscure the topology of strong and significant connections (Rubinov and Sporns, NeuroImage 2010). Therefore, we applied a density thresholding strategy to obtain the relatively stable functional connectivity patterns, which is a traditional approach for the functional network construction (Bullmore and Bassett, Annu Rev Clin Psychol 2011; Liao et al., Neurosci Biobehav R 2017). Negative correlations were not included due to their controversial physiological interpretations (Fox et al., J Neurophysiol 2009; Murphy and Fox, NeuroImage 2017).

We further assessed the robustness of developmental effects on nodal entropy by considering three other network thresholding strategies, including 10%, 20%, and 30%. Under these conditions, the spatial patterns of age effects on nodal entropy remained highly similar to the main results (Figure S4). In the revised manuscript, we have briefly discussed this issue and provided additional analyses.

The relevant Method section (Page 24):

“To assess the robustness of the developmental effects of node entropy, we examined the potential impact of the network thresholding strategy, the statistical model for estimating age effects, and the head motion control strategy. First, as some weaker connections in functional networks play a key role in the development of human cognitive function [110], we examined age effects with a wider range of network thresholds, including 10%, 20%, and 30%. The latter threshold doubled the density of connecting edges used in the main results.”

The relevant Result section (Page 13):

“Subsequently, the development of nodal entropy was validated by employing different network thresholding strategies (i.e. 10%, 20%, and 30%) (Figure S4) and the generalized additive mixed model for estimating age effects (Figure S5). The spatial patterns of age effects on nodal entropy remained highly similar to the main results, with spatial correlations ranging from 0.70 to 0.98.”

The relevant Discussion section (Page 15):

“Of note, identifying the overlapping modular architecture using the current edge-centric method faces several challenges. First, uncertainties remain regarding whether and how the functional correlation matrix is thresholded to construct a stable node-level functional network [74, 75].”

The relevant Supplementary section (Page 36):

**Figure S4. Spatial patterns of developmental changes in nodal entropy for different thresholding strategies.** For each rsfMRI scan of children, the brain functional network was generated with different network thresholding strategies. **(A)** Network density of 10% for functional network construction. **(B)** Network density of 20% for functional network construction. **(C)** Network density of 30% for functional network construction. **(D)** Network density of 15% (i.e. main result as a reference). In each case, all the network construction and analysis strategies were set to be the same as those in the main analysis, except for the strategy of interest. All correlations were assessed using Pearson’s correlation across nodal regions. The significance of spatial similarity was assessed by comparing the observed value to a null distribution generated through 10,000 permutations that retained the spatial autocorrelation characteristics of the original age effect map (*t*-value map) in the main results.

**1.15** "To assess the statistical significance of the prediction accuracy, we generated a null distribution of accuracy using permutation tests (n = 100) by shuffling the actual entropy across nodes for each scan." Is n=100 correct? Looks like 10k in main text, and the p value for 7b indicates 10k permutations (because it can't be more precise than p<0.01 with 100 tests). If I'm simply misunderstanding, it would be great if the authors could clarify this.

**R:** In this study, we examined the significance level of the prediction accuracy based on the same null distribution for all subjects. Specifically, we first generated a null distribution of accuracy by shuffling nodal entropy across nodes for each scan (*n* = 100), and then aggregated the null distributions across all 446 scans. This aggregation results in a null distribution with 44,600 (i.e. 446 scans × 100 times) instances, which allows the *p*-value to be less than 0.01. In the revised manuscript, we have rephrased the descriptions of the generation of the null distribution.

The relevant Methods section (Page 23):

“To assess the statistical significance of the prediction accuracy, we generated a null distribution of prediction accuracy for the whole population using permutation tests. Specifically, we first generated a null distribution of accuracy by shuffling nodal entropy across nodes for each scan (*n* = 100), and then aggregated the null distributions across 446 scans, resulting in the final null distribution with 44,600 (i.e. 446 scans × 100 times) instances.”

**1.16** Further, it's not clear that leave-region-out is a valid approach due to spatial autocorrelation. Please use leave entire-subjects-out if you want to include this analysis, or explain to me why that is not possible/valid in this instance. In my opinion, univariate associations between entropy and structure metrics are more informative than multivariate regression here, particularly if the authors are trying to claim something mechanistic.

**R:** Thank you for these constructive suggestions. The validation approach and association analysis are addressed separately below.

First, we think that the leave-region-out and leave-entire-subject-out strategies address different questions of interest. The leave-region-out strategy primarily builds a subject-specific prediction model to explore brain features that contribute to the overall spatial pattern of nodal entropy, and the model may vary across individuals. In contrast, the leave-entire-subject-out strategy builds a node-specific prediction model to identify the brain features that contribute to individual variability in nodal entropy, and the prediction model and related features may vary across nodes. In this study, we focus on brain features that are relative to the overall inhomogeneous spatial pattern of nodal entropy, and thus used the leave-region-out approach. In addition, to address your concerns, we built the SVR models for each brain node separately. The prediction accuracy of nodal entropy remarkably varied across nodes, ranging from -0.14 – 0.14. Therefore, the leave-entire-subject-out strategy may not serve as an effective candidate model to capture the structural features related to nodal entropy.

**Figure. Spatial pattern of prediction accuracy of nodal entropy using structural brain features across subjects.** To identify structural features contributing to individual variability in nodal entropy, we built a linear support vector regression model for each brain node separately with ten-fold cross-validation. To avoid the possibility of data leakage that could occur by including longitudinal scans from the same subjects between both the training and test sets, we performed the ten-fold cross-validation using 305 rsfMRI scans that were selected from independent subjects. For children with longitudinal scans, only one scan was selected for each individual and the other scans were discarded. To reduce the potential bias, the ten-fold cross-validation analysis was repeated 1,000 times with random scan selection. The figure displays the mean prediction accuracy for each brain region over 1,000 times with random sampling.

Second, as you suggested, we additionally examined the spatial similarity between whole-brain nodal entropy and each structural feature within each scan using univariate correlation analysis. The proportion of scans showing a significant correlation with a structural feature varied from 5% to 46% across the six structural features. Of these significant associations, only cortical thickness showed a positive spatial correlation with nodal entropy, while the other structural features showed negative correlations (Figure S1). These results were consistent with the contribution weights obtained from the multivariate model (i.e. support vector regression) used in the main results. In the main results, we found that structural features significantly predicted individual spatial patterns of nodal entropy in 95% of scans (424/446), suggesting that the multivariate correlation may better explain the relationship between nodal entropy and structural features. In the revised manuscript, we have added the methods and results of the univariate association analysis.

The relevant Methods section (Page 23):

“Prior to the prediction analysis, we first examined the spatial similarity between nodal entropy and each structural feature of interest using a univariate association analysis (i.e. Pearson’s correlation). Then, we performed the prediction using the same framework as that used in the age prediction mentioned above by integrating multiple structural features.”

The relevant Results section (Page 11 and 12):

“For each brain node of each child, we obtained six structural features, including five morphological measurements (cortical volume, thickness, curvature, folding index, and surface area) using structural MRI data and one white matter microstructural measure (fractional anisotropy strength) using diffusion MRI data. We initially examined the spatial similarity between nodal entropy in the overlapping functional modules and each structural feature within each scan using the univariate Pearson’s correlation. Cortical thickness showed a positive correlation with nodal entropy, while the other structural features exhibited negative correlations (Figure S1). Next, the individual spatial pattern of nodal entropy was predicted by integrating all structural brain features using the SVR model.”

The relevant Supplementary materials section (Page 33):

**Figure S1. Univariate relationship in spatial patterns between nodal entropy and structural brain features within each scan in children.** For each scan, we separately calculated the Pearson’s correlation in spatial patterns between nodal entropy and each structural brain feature. **(A)** Frequency polygon of correlation coefficients for cortical thickness across rsfMRI scans. Compared to the null distribution, cortical thickness showed significant positive spatial correlations with nodal entropy in 17% of scans (74/446) (*p* < 0.05). **(B)** Frequency polygon of correlation coefficients for surface area in all rsfMRI scans. Compared to the null distribution, surface area showed significant negative correlations with nodal entropy in 44% of scans (196/446) (*p* < 0.05). **(C)** Frequency polygon of correlation coefficients for folding index in all rsfMRI scans. Compared to the null distribution, folding index showed significant negative correlations with nodal entropy in 46% of scans (207/446) (*p* < 0.05). **(D)** Frequency polygon of correlation coefficients for FA strength in all rsfMRI scans. Compared to the null distribution, FA strength showed significant negative correlations with nodal entropy in 5% of scans (24/446) (*p* < 0.05). **(E)** Frequency polygon of correlation coefficients for cortical curvature in all rsfMRI scans. Compared to the null distribution, cortical curvature showed significant negative correlations with nodal entropy in 30% of scans (136/446) (*p* < 0.05). **(F)** Frequency polygon of correlation coefficients for cortical volume in all rsfMRI scans. Compared to the null distribution, cortical volume showed significant negative correlations with nodal entropy in 17% of scans (76/446) (*p* < 0.05). In (A)-(F), the inset in the upper corner denotes the null distribution of the correlation coefficients. This null distribution was generated by aggregating the 100 permutation instances for each scan, resulting in a total of 44,600 permutation instances (446 scans × 100 times). For each permutation within each scan, a surrogate nodal entropy map was generated that preserved the spatial autocorrelation characteristics of the original nodal entropy map [56]. The red line denotes the 95% significance level in the null distribution.

**Reviewer #2:**

In this work, the authors use a large longitudinal sample of children ages 6-14 years of age to look at how overlapping module assignment in edge-centric functional networks varies with age. They find that areas that show high and low overlap in assignment are relatively stable with age, with higher overlap in VA and SM networks and lower in VIS and DM. Some particular regions show age-related changes, such that their module assignment becomes more (PFC) or less (parietal cortex) stable with age. The authors also conduct ML analyses using SVR to show that module assignment overlap can predict age, and that regional variation in module overlap is associated most strongly with regional variation in patterns of cortical thickness.

The study has several strengths, including a large longitudinal sample and novel methods, and I very much appreciate the set of validation analyses. There are some weaknesses, specifically around clarity and interpretation of findings as well as methodological assumptions, that could be remedied. I've included specific comments on these aspects below.

**R:** We thank you for the positive comments and insightful suggestions.

**Major comments**

**2.1** Intro is well-motivated for a study focusing on soft partitioning of functional networks and overlap of network assignments…but needs more motivation/ explanation as to why taking an edge-centric approach in addition to a soft partitioning approach is necessary. It seems the authors are taking two steps from what has previously been done, rather than the one that they motivate in the intro, which makes comparison to previous studies harder.

**R:** Thank you for pointing out this issue. In this study, we highlight that soft partitioning of module affiliations of brain regions provides novel insights into the diverse functional roles of brain regions. And the edge-centric approach was used to achieve soft partitioning at the region level. Specifically, the multiple module affiliations of each brain region were obtained by simultaneously assigning the module affiliations of all its edges, while the module affiliations of edges were identified by hard partitioning in the corresponding edge graph. In the revised manuscript, we have clarified this issue in the Introduction section.

The relevant Introduction section (Page 4):

“In the present study, we identified overlapping functional modules using an edge-centric module detection approach [44], which makes no assumptions about the probabilistic membership of nodes. This approach provides an intuitive way to assign nodes to various communities based on the module affiliations of their edges (referred to as nodal soft partitioning) [45]. The overlapping extent of each brain node in the module affiliations was quantified with an entropy measure [34].”

**2.2** Similarly, in the discussion, the authors frame their findings in the context of previous work on overlapping functional modular organization, but don't mention how their methods differ significantly (in the focus on edges) from other prior work that used nodal FC but characterized overlapping modular architecture.

**R:** Thank you for this valuable suggestion. We have added a brief discussion in the revised manuscript.

The relevant Discussion section (Page 15):

“Previous studies on overlapping modules in the human brain have primarily used node-centric methods [32, 33, 35, 39], usually assuming probabilistic membership or maximal clique for nodes, which struggle to capture intricate overlapping configuration [72]. In this study, we employed an edge-centric algorithm [30, 44] to identify the overlapping modular architecture for brain nodes, offering an intuitive and detailed representation of multiple module affiliations of nodes [30, 34]. A recent study suggests that the overlapping modular architecture revealed by the edge-centric approach can be explained by surrogate interedge similarity derived from the node-level functional connectivity [73]. Further research is needed to explore the differences between these two approaches.”

**2.3** There has been some work using soft partitioning methods that capture overlap in the age range studied that is not covered in the intro. Tooley et al 2022 (Neuroimage) and Faskowitz et al. 2018 (Sci Reports) come to mind. Dworetsky et al. 2021 (Neuroimage) and Hermosillo 2024 (Nat Neuro) might also be relevant, though in adults.

**R:** Thank you for these valuable suggestions. In the revised manuscript, we have cited the relevant references in the appropriate sections.

The relevant Discussion section (Page 14, 16):

“Some recent studies have identified fuzzy network affiliations of brain regions in children using the clustering or template matching approaches, but ignoring their developmental changes at this stage [36, 64].”

“However, the spatial distribution of the overlapping modules in functional networks does not align consistently with that in structural networks [44, 80]. Further work is needed to elucidate how structural brain networks shape functional overlapping modules during development by establishing computational network models, such as communication models [81] and large-scale dynamic modeling [82].”

**2.4** The reader needs clearer explanation of what goes into constructing a weighted edge graph, and perhaps an addition to Fig 1. Given that an edge represents the similarity of fluctuations in BOLD signal between two regions, what does the weighted edge graph represent? What is being evaluated for similarity between edges? I had to go look at Faskowitz 2020 to remind myself.

**R:** In this study, we constructed the edge graph using the method proposed by Ahn and colleagues (Nature, 2010), which differs from the approach proposed by Faskowitz and colleagues (Nat Neurosci, 2020). Each link in the weighted edge graph denotes the similarity between edges in the connectivity profiles. For simplicity and computational efficiency, we only considered directly connected edge pairs that shared a common node, which captures the direct and dominant interaction between edges, as proposed by Ahn and colleagues (Nature, 2010). The similarity between edges without common nodes was assumed to be zero. In the revised manuscript, we have provided further explanations and modified the schematic diagram (Figure 1) to better illustrate the procedure for edge graph construction.

The relevant Methods section (Page 18 and 19):

“*Edge graph construction.* For each functional network, we constructed a corresponding weighted edge graph that denoted the similarity between edges (Figure 1B). For simplicity and computation efficiency, we considered only directly connected edge pairs that shared one common node based on the hypothesis proposed in Ahn and colleagues [30], which captures the direct and dominant interaction between edges. The similarity between edges without common nodes was assumed to be zero. The Tanimoto coefficient [30, 46] was used to incorporate the edge weight information. For a pair of edges *e_ik_* and *e_jk_* that share a common node *k*, their similarity was defined based on the similarity in the connection profiles between nodes *i* and *j.*”

The relevant Results section (Page 5):

**Figure 1 (B)** (i) Traditional brain functional connectivity network. In this network, each node denotes a brain region of interest, and each link denotes the interregional functional connectivity. (ii) Edge graph corresponding to a given functional network. In this graph, each node denotes an edge in the functional network, and each link is defined as the similarity between edges in the connectivity profiles using Tanimoto coefficient [30, 46]. For two given edges *e_ik_* and *e_jk_* that share a common node *k*, the interedge similarity was estimated as the similarity of connectivity profiles between node *i* and node *j*, wherein *a_i_* represents the modified connectivity profile of node *i*, $a_{i}\cdot a_{j}$ represents the dot product of two vectors *a_i_* and *a_j_*, and |*a_i_*|^2^ denotes the sum of the squared weights of all connections of node *i*. (iii) Edge-centric module detection. Each edge is assigned to a specific module based on the Louvain algorithm [47]. (iv) Definition of regional module overlap. Each nodal region was assigned to one or more modules due to the diverse module affiliations of its edges. A measure of entropy was employed to quantify the extent of module overlap of each brain node by measuring the distribution of the module affiliations of its edges [34].

**2.5** Thresholding at an arbitrary density is problematic for interpretation of results. Other work using edge-centric methods does not necessitate a specific threshold or the removal of negative edges before constructing an weighted edge matrix. This simply indicates that all results are likely based on only examining the strongest connections, while there is good evidence that weak connections might be changing during development and play an important role. The authors should conduct supplemental analyses using fully weighted matrices and/or matrices at a wider variety of densities to investigate this.

**R:** Thank you for raising this issue. In the edge-centric approach, the estimation of module affiliations of a brain node depends on the module assignment of all the edges of that node. Thus, the accurate and stable estimation of the edges of interest and the inter-edge association is crucial for the subsequent overlapping modules analysis. In the work of Faskowitz and colleagues (2020), the edge graph is indeed constructed for all possible pairs of edges, but the reliability of the estimation is affected by the rsfMR scan duration. To reduce potential influence of rsfMRI scan duration, we used a different edge-centric approach (Ahn et al., Nature 2010) to construct the edge graph based on the static functional network, as functional correlations tend to stabilize after 5-6 minutes (Van Dijk et al., J Neurophysiol 2010; Whitlow et al., Radiology 2011). Furthermore, previous studies suggest that weak and non-significant functional correlations may represent spurious connections and tend to obscure the topology of strong and significant connections (Rubinov and Sporns, NeuroImage 2010). Therefore, we applied a density thresholding strategy to obtain relatively stable functional connectivity patterns, which is a traditional approach for the functional network construction (Bullmore and Bassett, Annu Rev Clin Psychol 2011; Liao et al., Neurosci Biobehav R 2017). Negative correlations were not included due to their controversial physiological interpretations (Fox et al., J Neurophysiol 2009; Murphy and Fox, NeuroImage 2017).

We further assessed the robustness of the developmental effects on nodal entropy by considering three other network thresholding strategies, including 10%, 20%, and 30%, respectively. Under these conditions, the spatial patterns of age effects on nodal entropy remained highly similar to the main results (Figure S4). In the revised manuscript, we have briefly discussed this issue and provided additional analyses.

The relevant Method section (Page 24):

“To assess the robustness of the developmental effects of node entropy, we examined the potential impact of the network thresholding strategy, the statistical model for estimating age effects, and the head motion control strategy. First, as some weaker connections in functional networks play a key role in the development of human cognitive function [110], we examined age effects with a wider range of network thresholds, including 10%, 20%, and 30%. The latter threshold doubled the density of connecting edges used in the main results.”

The relevant Result section (Page 13):

“Subsequently, the development of nodal entropy was validated by employing different network thresholding strategies (i.e. 10%, 20%, and 30%) (Figure S4) and the generalized additive mixed model for estimating age effects (Figure S5). The spatial patterns of age effects on nodal entropy remained highly similar to the main results, with spatial correlations ranging from 0.70 to 0.98.”

The relevant Discussion section (Page 15):

“Of note, identifying the overlapping modular architecture using the current edge-centric method faces several challenges. First, uncertainties remain regarding whether and how the functional correlation matrix is thresholded to construct a stable node-level functional network [74, 75].”

The relevant Supplementary section (Page 36):

**Figure S4. Spatial patterns of developmental changes in nodal entropy for different thresholding strategies.** For each rsfMRI scan of children, the brain functional network was generated with different network thresholding strategies. **(A)** Network density of 10% for functional network construction. **(B)** Network density of 20% for functional network construction. **(C)** Network density of 30% for functional network construction. **(D)** Network density of 15% (i.e. main result as a reference). In each case, all the network construction and analysis strategies were set to be the same as those in the main analysis, except for the strategy of interest. All correlations were assessed using Pearson’s correlation across nodal regions. The significance of spatial similarity was assessed by comparing the observed value to a null distribution generated through 10,000 permutations that retained the spatial autocorrelation characteristics of the original age effect map (*t*-value map) in the main results.

**2.6** "For simplicity, we considered directly connected edges that shared at least one common node, and the similarity between edges without common nodes was assumed to be zero. " This seems like an extremely strong assumption, and not one that I'm familiar with in other work. No rationale is provided or supplemental analyses showing that this is a reasonable assumption to make are included.

**R:** In this study, we estimated the inter-edge similarity primarily based on the hypothesis proposed by Ahn and colleagues (Nature, 2010). Ahn et al. (2010) argued that edge pairs without common nodes are unlikely to show stronger associations than those with common nodes. Therefore, the edge-graph construction is limited to directed connected edge pairs, capturing the dominant relationship between edges. Our analysis follows this hypothesis. In the revised manuscript, we have added some explanations to clarify this issue.

The relevant Methods section (Page 18):

“For simplicity and computation efficiency, we considered only directly connected edge pairs that shared one common node based on the hypothesis proposed in Ahn and colleagues [30], which captures the direct and dominant interaction between edges. The similarity between edges without common nodes was assumed to be zero.”

The relevant Discussion section (Page 15):

“Second, this study only considers the relationships between edge pairs that share common nodes, neglecting potential indirect relationships between edges. Higher-order relationships between edges could be considered in future studies.”

**2.7** Is the Tanimoto coefficient a typical way to assess edge similarity? I see no mention of it in Faskowitz et al (2020). Or is this paper also introducing a different way of assessing edge similarity than has been previously used? If so, why?

**R:** The Tanimoto coefficient is a typical way to measure the similarity between two sets. In this study, we used it to estimate inter-edge similarity (Ahn et al., Nature 2010), which is different from the method used in Faskowitz et al. (2020). Faskowitz and colleagues defined inter-edge similarity based on the time-resolved co-fluctuations of edge time series, but the reliability of this approach is affected by the rsfMRI scan duration. To reduce the potential influence of scan duration, we constructed the edge graph based on the static functional network, as functional correlations generally stabilize after 5-6 minutes (Van Dijk et al., J Neurophysiol 2010; Whitlow et al., Radiology 2011). We then calculated the inter-edge similarity using the Tanimoto coefficient to quantify the similarity of the connection profiles between two unshared nodes of the edges (Figure 1B).

**2.8** If the main paper finding is related to the nodal entropy map (which the validation analyses suggest is the case), I'd suggest emphasizing the interpretation of this specific finding in the abstract and first paragraph of discussion in a clearer way. Findings that there are decreases in nodal overlap in module affiliations in PFC imply what for brain development? To my mind, it implies that PFC is becoming more homogenously affiliated, or more stable/less flexible in its network affiliation, with age. Some phrases of this nature would be helpful for a reader not versed in soft-partitioning methods.

**R:** In the revised manuscript, we have added some explanations in the relevant paragraphs.

The relevant Abstract section (Page 2):

“The overlapping modules developed in a linear, spatially dissociable manner, with decreased entropy (i.e. decreased module involvement) in the dorsomedial prefrontal cortex, ventral prefrontal cortex, and putamen and increased entropy (i.e. increased module involvement) in the parietal lobules and lateral prefrontal cortex.”

The relevant Discussion section (Page 13):

“The nodal modular overlap (i.e. nodal entropy) developed with remarkable spatial heterogeneity, with significant decreases in the ventral and medial prefrontal cortex and putamen and significant increases in the superior and inferior parietal lobules and the lateral prefrontal cortex. These changes suggest an age-related reorganization in intermodule integration.”

**Minor comments**

**2.9** Some clearer takeaways in the abstract might be appreciate by readers (e.g. what does regional inhomogeneity in module overlap mean for children's brain networks?)

**R:** We have rephrased the abstract and added an explanation of "regional inhomogeneity in module overlap" in the abstract.

The relevant Abstract section (Page 2):

“We showed a regionally heterogeneous spatial topography of the overlapping extent of brain nodes in module affiliations in children, with higher entropy (i.e. more module involvement) in the ventral attention, somatomotor, and subcortical regions and lower entropy (i.e. less module involvement) in the visual and default-mode regions.”

**2.10** The values of edge correlations in Fig 2a seem quite low, compared to the corresponding figure in Faskowitz 2020, and this figure further reinforces that setting many edge similarities to 0 without calculating them may be a strong assumption.

**R:** Thank you for pointing out this issue. In this study, the edge-centric approach used to identify the overlapping modular architecture is different from the approach used in Faskowitz et al. 2020. Therefore, the inter-edge similarity values are not directly comparable. For simplicity and efficiency, we focused on the edge pairs that shared at least one common node based on the hypothesis proposed by Ahn et al. (Nature, 2010), and the similarity between edges without common nodes was assumed to be zero. The higher order relationship between edges can be explored in the future by developing more sophisticated measures. In the revised manuscript, we have explained these issues in the relevant sections.

The relevant Methods section (Page 18):

“For simplicity and computation efficiency, we considered only directly connected edge pairs that shared one common node based on the hypothesis proposed in Ahn and colleagues [30], which captures the direct and dominant interaction between edges. The similarity between edges without common nodes was assumed to be zero.”

The relevant Discussion section (Page 15):

“Second, this study only considers the relationships between edge pairs that share common nodes, neglecting potential indirect relationships between edges. Higher-order relationships between edges could be considered in future studies.”

**2.11** Would Fig 1d be better off as a graph with differently colored or weighted lines? It is initially difficult to interpret what the reader is trying to understand from the colors and sizes of circles.

**R:** As there is no part d in Figure 1, we speculate that you might refer to Figure 2d. In the revised manuscript, we have updated the Figure 2d as colored lines as follows.

The relevant Results section (Page 5):

“In general, most functional modules in the child subgroups showed high spatial similarity with those in the adult subgroup (Pearson’s correlation *r*s: mean ± SD = 0.75 ± 0.02, range: 0.25-0.95) (Figure 2D). Initial inspection revealed that the spatial similarity with the adult group tended to increase with age for all modules, except modules V and VI.”

**“Figure 2. Overlapping modular architecture of group-level brain functional networks in the adult cohort and the child subgroups. … (D)** Spatial similarity of functional module maps between the child subgroups and the adult group. Each line represents the age-dependent similarity for a particular module.”

**2.12** It is quite interesting that looking at Fig 3b and comparing to adults, it seems that the overall trend might be decreasing entropy with age (both in insular areas and subcortex); this would imply that module assignments based on edge similarity are more stable in adults than children. Did the authors examine average whole-brain entropy (or cortical vs subcortical) associations with age?

**R:** According to your suggestion, we also examined the age effects on the mean entropy of the whole brain, the cortical nodes, and the subcortical nodes. We found that whole-brain mean entropy (*t* = -1.54, *p* = 0.12) and cortical mean entropy (*t* = -0.24, *p* = 0.81) did not show significant correlations with age. However, subcortical mean entropy showed a significant decrease with age (*t* = -3.13, *p* = 0.0019), as shown in the system-level analysis (Figure 4B).

The relevant Results section (Page 7):

“The global entropy (i.e. mean nodal entropy across the brain) did not show significant correlation with age (*t* = -1.54, *p* = 0.12).”

“Quantitative analysis revealed that the entropy of the dorsal attention system significantly increased with age (linear model, *t* = 2.44, *p* = 0.015), while the entropy in the subcortical area significantly decreased with age (linear model, *t* = -3.13, *p* = 0.0019, Figure 4B).”

**2.13** The claim on page 13 line 27 seems quite strong, given some of the other research that exists already.

**R:** We have rephrased the relevant description in the revised manuscript.

The relevant Discussion section (Page 13):

“In this study, we used the novel framework of overlapping modular architectures, which provides an intuitive representation of the functional interactions between modules and the multiple roles of brain regions [59, 60] and deepens the understanding of the organizational principles of the human brain [31-35]. Compared to previous studies on overlapping modules in adults [31-35] and the elderly [40], our research focuses on the development of overlapping functional modular organization during childhood and adolescence.”

**2.14** I'd find the title easier to parse as "Development of overlapping network modules in the human brain". I would be more specific than "signatures" when describing associations with brain structure.

**R:** In the revised manuscript, the title has been changed to "Development of Overlapping Network Modules in the Human Brain". The terms of "signatures" have been rephrased as "gray and white matter features" throughout the manuscript.
