# Supplementary material for: Functional network modules overlap and are linked to interindividual connectome differences during human brain development
Source: PLoS Biol. 2024 Sep 18;22(9):e3002653. doi: 10.1371/journal.pbio.3002653 (PMC11441662; doi:10.1371/journal.pbio.3002653)
Supplement: Attachment — Submitted filename: ResponsesToReviewers.docx [file pbio.3002653.s008.docx]

**Reviewer #1:**

The authors demonstrated a strong effort in addressing our comments. I am convinced of their findings and commend their work. I also appreciate them clarifying and explaining several points to me.

I'm suggesting acceptance, but here are two remaining minor points for the authors to consider.

**R:** Thank you for your positive comments and further suggestions.

1. The caption for Figure 6B isn't clear. The gray frequency polygon is referenced, but not the blue one. I assume the first sentence after (B) refers to blue, but simply adding an equivalent sentence structure for the blue frequency polygon would make the difference between what they are intended to display more clear.

**R:** In the revised manuscript, we have revised the caption for Figure 6B to improve readability.

The relevant Results section (Page 11):

“**(B)** Frequency polygon for age prediction accuracy using the ten-fold SVR model for 1,000 times of randomly selected samples (blue histogram). For each time of random sampling, rsfMRI scans were randomly selected from 305 independent subjects. The gray frequency polygon in the inset displays the null distribution of prediction accuracy based on the permutation tests (*n* = 10,000) by randomly sampling the scans and shuffling the original ages across the scans. The red line in the blue histogram indicates the significant level (*p* < 0.05) derived from the null distribution.”

2. I'm not a fan of displaying only p-values from models as they have done in figure S5. Because it's a supplementary analysis and because GAMMs don't have straightforward coefficients, it's not a deal-breaker as is. But I would avoid doing that in future work.

**R:** Since GAMMs do not provide straightforward coefficients, we had to opt for this approach. However, we appreciate your suggestion and will avoid using this method in future work.
